# Multi-omics approaches to disease

**DOI:** 10.1186/s13059-017-1215-1

**Published:** 2017-05-05

**Authors:** Yehudit Hasin, Marcus Seldin, Aldons Lusis

**Affiliations:** 1 Department of Medicine, University of California, 10833 Le Conte Avenue, A2-237 CHS, Los Angeles, CA 90095 USA; 2 Department of Microbiology, Immunology and Molecular Genetics, 10833 Le Conte Avenue, A2-237 CHS, Los Angeles, CA 90095 USA; 3 Department of Human Genetics, University of California, 10833 Le Conte Avenue, A2-237 CHS, Los Angeles, CA 90095 USA

## Abstract

High-throughput technologies have revolutionized medical research. The advent of genotyping arrays enabled large-scale genome-wide association studies and methods for examining global transcript levels, which gave rise to the field of “integrative genetics”. Other omics technologies, such as proteomics and metabolomics, are now often incorporated into the everyday methodology of biological researchers. In this review, we provide an overview of such omics technologies and focus on methods for their integration across multiple omics layers. As compared to studies of a single omics type, multi-omics offers the opportunity to understand the flow of information that underlies disease.

## Introduction

The addition of “omics” to a molecular term implies a comprehensive, or global, assessment of a set of molecules (http://omics.org/). The first omics discipline to appear, genomics, focused on the study of entire genomes as opposed to “genetics” that interrogated individual variants or single genes. Genomic studies provided a very useful framework for mapping and studying specific genetic variants contributing to both mendelian and complex diseases. The omics field has been driven largely by technological advances that have made possible cost-efficient, high-throughput analysis of biologic molecules. For example, the “expression array”, based on hybridization of cDNA to arrays of oligonucleotide capture probes, was developed in the late 1990s. With refinement, array technologies proved capable of quantifying the levels of all protein coding transcripts in a particular tissue. The ability to survey global gene expression patterns quickly found application in many fields of biology, including the analysis of disease. In the early 2000s, array technologies also made it possible to map loci that control gene expression, termed expression quantitative trait loci (eQTL), which have proved invaluable in the interpretation of genome-wide association studies (GWAS) and the modeling of biologic networks. Since then, many other omics technologies have been developed that are capable of interrogation of entire pools of transcripts, proteins, and metabolites, as well as the genome (Box 1).

**Table:** 

**Box 1. Omics data types**
**Genomics** is the most mature of the omics fields. In the realm of medical research, genomics focuses on identifying genetic variants associated with disease, response to treatment, or future patient prognosis. GWAS is a successful approach that has been used to identify thousands of genetic variants associated with complex diseases (GWAS catalog https://www.ebi.ac.uk/gwas/home) in multiple human populations. In such studies, thousands of individuals are genotyped for more than a million genetic markers, and statistically significant differences in minor allele frequencies between cases and controls are considered evidence of association. GWAS studies provide an invaluable contribution to our understanding of complex phenotypes. Associated technologies include genotype arrays [111–114], NGS for whole-genome sequencing [115, 116], and exome sequencing [117]. **Epigenomics** focuses on genome-wide characterization of reversible modifications of DNA or DNA-associated proteins, such as DNA methylation or histone acetylation. Covalent modifications of DNA and histones are major regulators of gene transcription and subsequently of cellular fate [118]. Those modifications can be influenced both by genetic and environmental factors, can be long lasting, and are sometimes heritable [119–121]. While the role of epigenetic modifications as mediators of transgenerational environmental effects remains controversial [122, 123], their importance in biological processes and disease development is evident from many epigenome-wide association studies that have been reported. For example, differentially methylated regions of DNA can be used as indicators of disease status for metabolic syndrome [124, 125], cardiovascular disease [126], cancer [127], and many other pathophysiologic states [128]. Epigenetic signatures are often tissue-specific [129], and several large consortia are focusing on establishing comprehensive epigenomic maps in multiple human tissues (Roadmap Epigenomics (http://www.roadmapepigenomics.org/) and International Human Epigenome Consortium (http://ihec-epigenomes.org/)). Thus, in addition to insight gained from identifying epigenetic modifications correlating with diseases, data generated by these studies has great potential to enhance our functional interpretation of genetic variants residing in those regions or of epigenetic markers associated with disease independently of genetic variation ([130] and other Roadmap Epigenomics publications). Associated technology includes assessment of DNA modifications using NGS [130]. **Transcriptomics** examines RNA levels genome-wide, both qualitatively (which transcripts are present, identification of novel splice sites, RNA editing sites) and quantitatively (how much of each transcript is expressed). The central dogma of biology viewed RNA as a molecular intermediate between DNA and proteins, which are considered the primary functional read-out of DNA. Other examples of RNA function, such as structural (e.g., ribosomal complexes), or regulatory (e.g., Xist in ChrX inactivation) have often been regarded as odd exceptions to the general rule. The advent of large transcriptomic studies in the past decade has shown that while only ~3% of the genome encodes proteins, up to 80% of the genome is transcribed [131]. RNA-Seq studies identified thousands of novel isoforms and showed a larger than previously appreciated complexity of the protein-coding transcriptome [132]. However, an even more significant contribution of these studies was the development of the non-coding RNA field. It is now clear that thousands of long non-coding RNAs transcribed in mammalian cells (http://www.gencodegenes.org/) play essential roles in many physiological processes, for example, brown adipose differentiation [133], endocrine regulation [134], and neuron development [135]. Dysregulation of long non-coding RNAs had been implicated in various diseases, such as myocardial infarction [136], diabetes [137, 138], cancer [139], and others [140]. In addition to long non-coding RNA, NGS allows interrogation of short RNAs (microRNAs, piwi-interacting RNAs, and small nuclear RNAs) and identification of circular RNAs, a novel player in the family of RNAs [141]. Much like long non-coding RNAs, a growing body of evidence points to dysregulation of short and circular RNAs in disease [142–144] and the potential use thereof as biomarkers or as therapeutic targets. Associated technologies include probe-based arrays [145, 146] and RNA-Seq [147, 148]. **Proteomics** is used to quantify peptide abundance, modification, and interaction. The analysis and quantification of proteins has been revolutionized by MS-based methods and, recently, these have been adapted for high-throughput analyses of thousands of proteins in cells or body fluids [149, 150]. Interactions between proteins can be detected by classic unbiased methods such as phage display and yeast two-hybrid assays. Affinity purification methods, in which one molecule is isolated using an antibody or a genetic tag, can also be used. MS is then used to identify any associated proteins. Such affinity methods, sometimes coupled with chemical crosslinking, have been adapted to examine global interactions between proteins and nucleic acids (e.g., ChIP-Seq). Finally, the functions of a large fraction of proteins are mediated by post-translational modifications such as proteolysis, glycosylation, phosphorylation, nitrosylation, and ubiquitination [151, 152]. Such modifications play key roles in intracellular signaling, control of enzyme activity, protein turnover and transport, and maintaining overall cell structure [153]. MS can be used to directly measure such covalent modifications by defining the corresponding shift in the mass of the protein (in comparison to the unmodified peptide). There are efforts to develop genome-level analyses of such modifications [154]. Associated technologies include MS-based approaches to investigate global proteome interactions and quantification of post-translational modifications [155, 156]. **Metabolomics** simultaneously quantifies multiple small molecule types, such as amino acids, fatty acids, carbohydrates, or other products of cellular metabolic functions. Metabolite levels and relative ratios reflect metabolic function, and out of normal range perturbations are often indicative of disease. Quantitative measures of metabolite levels have made possible the discovery of novel genetic loci regulating small molecules, or their relative ratios, in plasma and other tissues [157–160]. Additionally, metabolomics in combination with modeling has been used extensively to study metabolite flux. Associated technologies include MS-based approaches to quantify both relative and targeted small molecule abundances [161–166]. **Microbiomics** is a fast-growing field in which all the microorganisms of a given community are investigated together. Human skin, mucosal surfaces, and the gut are colonized by microorganisms, including bacteria, viruses, and fungi, collectively known as the microbiota (and their genes constituting the microbiome). The human microbiome is enormously complex; for example, the gut contains roughly 100 trillion bacteria from 1000 different species. There are substantial variations in microbiota composition between individuals resulting from seed during birth and development, diet and other environmental factors, drugs, and age [33]. Many studies have implicated perturbations in gut bacteria in a variety of disorders, including diabetes, obesity, cancer, colitis, heart disease, and autism. The microbiome can be profiled by amplifying and then sequencing certain hypervariable regions of the bacterial 16S rRNA genes followed by clustering the sequences into operational taxonomic units. Shotgun metagenomics sequencing, in which total DNA is sequenced, can provide additional resolution for distinguishing genetically close microbial species. Several analytic tools have been developed for analyzing NGS data from targeted 16S or metagenomics analysis, such as QIIME (quantitative insights into microbial ecology) [167]. These allow accurate quantitative determination of taxa that can be correlated with disease or other phenotypes of interest [168]. Associated technologies include NGS application for 16S ribosomal abundance and metagenomics quantification [169–172].

In the past decade, high-throughput genotyping, combined with the development of a high quality reference map of the human genome, rigorous statistical tools, and large coordinated cohorts of thousands of patients, has enabled the mapping of thousands of genetic variants, both rare and common, contributing to disease [1–3]. However, as our power to identify genetic variants associated with complex disease increased several realizations were reached that have shaped subsequent approaches to elucidating the causes of disease. First, the loci that have been identified so far generally explain only a fraction of the heritable component for specific diseases. Second, while Mendelian diseases generally result from changes in coding regions of genes, common diseases usually result from changes in gene regulation. Third, the same genetic variants often contribute to different final outcomes, depending on the environment and genetic background. Taken together, these realizations provided a rationale for the development of systems biology technologies that involve the integration of different omics data types to identify molecular patterns associated with disease.

Each type of omics data, on its own, typically provides a list of differences associated with the disease. These data can be useful both as markers of the disease process and to give insight as to which biological pathways or processes are different between the disease and control groups. However, analysis of only one data type is limited to correlations, mostly reflecting reactive processes rather than causative ones. Integration of different omics data types is often used to elucidate potential causative changes that lead to disease, or the treatment targets, that can be then tested in further molecular studies.

In this review, we focus on the integration of multiple types of omics data (“multi-omics” or “vertical omics”) as applied to research on human disease. This review is divided into three sections. First, we outline considerations that apply to experimental design and collection of omics data. Second, we discuss general frameworks for integration of omics data in disease research and outline analytic strategies. Finally, we speculate about the future directions of multi-omics approaches.

## Considerations for the design of omics studies

**Fig. 1:**
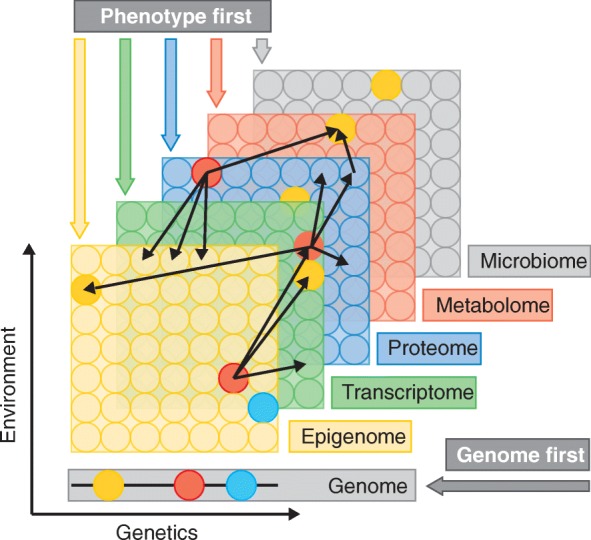
Multiple omics data types and approaches to disease research. Layers depict different types of omics data (Box 1). Omics data are collected on the entire pool of molecules, represented as *circles*. Except for the genome, all data layers reflect both genetic regulation and environment, which may affect each individual molecule to a different extent. The *thin red arrows* represent potential interactions or correlations detected between molecules in different layers—for example, the red transcript can be correlated to multiple proteins. Within layer interactions, although prevalent, are not depicted. *Thicker arrows* indicate different potential starting points or conceptual frameworks for consolidating multiple omics data to understand disease. The genome first approach implies that one starts from associated locus, while the phenotype first approach implies any other layer as the starting point. The environment first approach (not shown) examines environmental perturbations

Compared to single omics interrogations (Box 1, Fig. 1), multi-omics can provide researchers with a greater understanding of the flow of information, from the original cause of disease (genetic, environmental, or developmental) to the functional consequences or relevant interactions [4, 5]. Omics studies, by their nature, rely on large numbers of comparisons, tailored statistical analyses, and a considerable investment of time, skilled manpower, and money. Therefore, careful planning and execution are required. In this section, we discuss general experimental parameters that should be considered when planning an omics study.

### Complexity of disease etiology

An important consideration in the design of a multi-omic study is the nature of the disorder. Simple diseases, arising from single gene mutations, involve few etiological factors, and those factors typically play deterministic roles in disease development, although the severity or progression of many diseases is affected by “modifier genes” or environmental factors. For example, the most common cause of cystic fibrosis is a single chloride channel mutation, enabling disease-related work to focus on the function of this gene [6]. Thus, concentrated omics efforts at specific time points, focusing on immediate molecular changes induced by the causative factor, are expected to produce sufficient insight to promote understanding of potential therapeutic strategies. Note that the prominent etiological factor does not have to be genetic and could, for example, be an infectious agent.

The etiology of complex diseases is far more intricate and is not centered on one specific factor. Different combinations of a variety of factors could converge into phenotypically similar states. Moreover, in the absence of a clear deterministic factor that induces the disease, results from a single layer of data are always associative and, because reactive effects usually outnumber the causative effects in biologic cascades, should be interpreted as such. Additionally, given that most common, complex diseases develop over time and involve both environmental and genetic factors, full mechanistic insight will require coordinated sets of several omics data at multiple time points, collected from many disease relevant tissues.

### Downstream analysis, sample sizes, and power

Omics approaches generate data to provide biological insight based on statistical inference from datasets that are typically large. As such, the power to detect associations or the flow of information strongly depends on effect size, heterogeneity of the background noise, and sample size, with the latter often being the only parameter controlled by researchers. Unfortunately, human studies are affected by a multitude of confounding factors that are difficult or impossible to control for (e.g., diet and lifestyle choices). Thus, the ability of omics approaches to produce meaningful insight into human disease is very much dependent on available sample sizes, and in many settings an underpowered study may not only be a shot in the dark, missing true signals, but it is also more likely to produce false positive results. This issue is well illustrated in the earlier days of candidate gene studies for complex diseases, where lack of appreciation of these factors led to many publications of non-reproducible genetic associations. An initial power calculation to ensure sufficient sample size and variation in outcomes is increasingly necessary in large-scale studies.

Another potential pitfall of omics approaches is insufficient attention to data analysis requirements, before and during data collection. General analytical pipelines for each type of omics data are available (Box 1); however, most omics fields have not yet developed an agreed gold standard. Moreover, these datasets are often large and complex, and require tailoring of the general statistical approach to the specific dataset. An important aspect of all omics study designs, to make sure that the collected data meet analysis requirements, is to envision the main goal of analysis and the analytical approach, before collecting the data. For example, a common consideration when planning RNA-Seq experiments would be the allocation of financial resources to balance the number of samples with depth of coverage. To identify differentially expressed genes between the cases and controls, the power provided by more samples is generally preferable to the increased accuracy provided by higher depth of sequencing. However, if the main purpose of the analysis is to identify new transcripts, or examine allele-specific expression, the higher depth of coverage is desirable [7–9] (https://genome.ucsc.edu/ENCODE/protocols/dataStandards/RNA_standards_v1_2011_May.pdf). In addition to financial limitations, data analysis should guide data collection to avoid or minimize technical artifacts, such as batch effects that could be introduced during all steps of sample processing and data acquisition [10–13]. In large studies, some technical artifacts cannot be avoided, and in these cases it is crucial to understand to what extent those artifacts limit our ability to draw conclusions from observations, and possibly introduce controls that would be able to quantify its effect.

### Human studies and animal models of disease

Both human and animal model omics studies provide important insight into disease. Humans are the main intended beneficiary of medical research, and naturally findings from human studies have greater translational potential than animal models. Several human centric consortia have produced a large body of transcriptomics and epigenomics data in multiple tissues, for example, the Roadmap Epigenomics Project (http://www.roadmapepigenomics.org/; Box 1) and GTEx (http://www.gtexportal.org/home/) analyzed epigenomic signatures and transcriptomics in dozens of human tissues and cell types. In addition, several large biobanks have been created to collect, store, and analyze thousands of human samples related to diseases. For example, the National Institute of Health and Care in Finland developed a network of biobanks across the country [14] to collect specimens and measurements from patients with different diseases. The UK biobank [15] collects samples and physiologic measures and follows 500,000 people with respect to their activity. These samples can be characterized with various omics approaches and used to identify molecular changes that occur during disease, or prior to it when prospective data are available.

While providing useful insight, human omics studies suffer from several limitations that can be addressed in animal studies only, provided the appropriate animal model of the disease is used. One could argue that primary human cell lines represent a suitable platform to explore disease without the need for animal models, and indeed cell lines have been used quite extensively to dissect detailed individual mechanistic pathways [16]. But their use is limited by the complex nature and convergence of multiple cell types causing most complex diseases. The advantages of using animal models include reproducibility, control of environmental factors, accessibility of relevant tissues, accurate phenotyping, availability of a virtually unlimited number of exact biological replicates, and the ability to experimentally follow up on hypotheses. Animal studies have been essential for examining the effects of environmental stressors such as responses to variation in diet, which often provide mechanistic insight into the relationship between omics data and the response to a stressor. Additionally, renewable populations of animal models, such as inbred strains of rats or mice, can be interrogated repeatedly and omics studies of such populations have led to the development of powerful datasets containing detailed omic, physiological, and pathological data collected under a variety of conditions [17–19]. Comparison of omics data between human and animal models can help validate biological relevance of the model itself, as was used in a recent study of Alzheimer’s disease (AD) [20]. Yet, animal models also have limitations. Many of the gene-specific models are limited to one genetic background, mouse models may not recapitulate the human biology of complex disease, and some manifestations of human disease can be difficult to test in the mouse model.

## Approaches to integrative analysis of multiple omics data

Multi-omics approaches have been applied to a wide range of biological problems and we have grouped these into three categories, “genome first”, “phenotype first”, and “environment first”, depending on the initial focus of the investigation. Thus, the genome first approach seeks to determine the mechanisms by which GWAS loci contribute to disease. The phenotype first approach seeks to understand the pathways contributing to disease without centering the investigation on a particular locus. And the environment first approach examines the environment as a primary variable, asking how it perturbs pathways or interacts with genetic variation. We then discuss briefly some statistical issues around data integration across omics layers and network modeling.

### The genome first approach

In the absence of somatic mutations, primary DNA sequence remains unaltered throughout life and is not influenced by environment or development. Thus, for disease-associated genetic variants, it is assumed that a specific variant contributes to, and is not a consequence of, disease. Such variants constitute a very powerful anchor point for mechanistic studies of disease etiology and modeling interactions of other omics layers. GWASs often identify loci harboring the causal variants, but lack sufficient power to distinguish them from nearby variants that are associated with disease only by virtue of their linkage to the causative variant. Moreover, the identified loci typically contain multiple genes, which from a genomic point of view could equally contribute to disease. Thus, although GWAS results may be immediately useful for risk prediction purposes, they do not directly implicate a particular gene or pathway, let alone suggest a therapeutic target. Locus-centered integration of additional omics layers can help to identify causal single nucleotide polymorphisms (SNPs) and genes at GWAS loci and then to examine how these perturb pathways leading to disease.

**Fig. 2:**
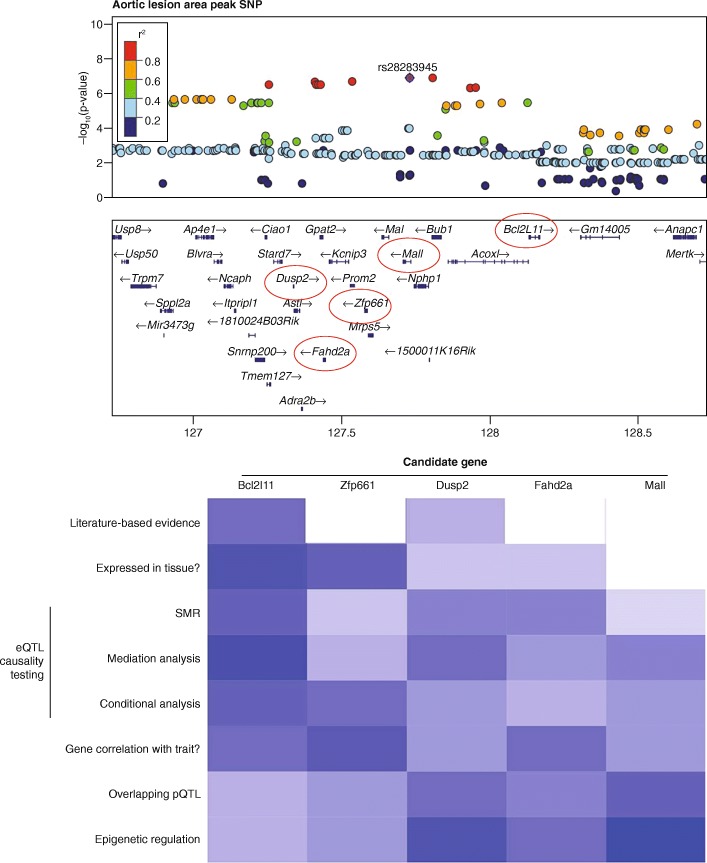
Usage of omics applications to prioritize GWAS variants. Locus zoom plot for a complex GWAS locus shows several candidate genes could be causal. Heatmap using various omics approaches for evidence supporting or refuting candidate causal genes. Beyond literature queries for candidates, various omics technologies and databases can be used to identify causal genes, including: searching for expression in relevant tissues [173–175], summary data-based Mendelian randomization (SMR) [176], mediation analysis [177], conditional analysis [23], correlation analyses, searching for overlapping pQTLs [178, 179], and/or implementing epigenetic data to narrow candidates (discussed for FTO locus [16])

Analyses of causal variants at GWAS loci focused originally on coding regions, but it has become clear that for many common diseases regulatory variation explains most of the risk burden [21]. Thus, transcriptomics, employing either expression arrays or RNA-Seq (Box 1), has proven to be particularly useful for identifying causal genes at GWAS loci [16, 22–24]. A number of statistical methods have been developed for examining causality based on eQTL at GWAS loci, including conditional analysis and mediation analysis (Fig. 2). Large datasets of eQTLs are now available for a number of tissues in humans and animal models [17, 22, 25, 26].

**Fig. 3:**
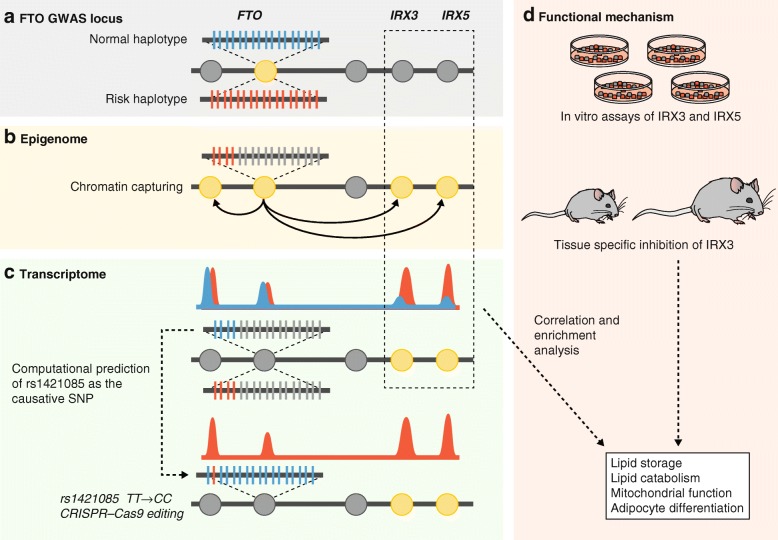
Genome first approach at FTO GWAS locus. Claussnitzer et al [16] combined genomics, epigenomics, transcriptomics, and phylogenetic analysis to identify the functional element, the causative SNP, and the downstream genes mediating the genetic effect at the FTO locus in obesity. *Circles* represent genes in the locus and *yellow circles* represent genes implicated by the respective omics data. **a** Genomics: the FTO locus, containing several genes (*circles*), harbors the most significant obesity-associated haplotype in humans. SNPs that are in linkage disequilibrium with the risk allele are color coded—*blue* represents the non-risk (normal) haplotype and *red* the risk haplotype. **b** Epigenomics: publically available epigenomic maps and functional assays were used to narrow down the original associated region to 10 kb containing an adipose-specific enhancer. Chromatin capturing (Hi-C) was used to identify genes interacting with this enhancer. **c** Transcriptomics: this technique was used to identify which of the candidate genes are differentially expressed between the risk and normal haplotypes, identifying IRX3 and IRX5 as the likely downstream targets. In addition, conservation analysis suggested that rs1421085 (SNP that disrupts an ARID5B binding motif) is the causative SNP at the FTO locus. CRISPR-Cas9 editing of rs1421085 from background (TT) to risk allele (CC) was sufficient to explain the observed differences in expression of IRX3 and IRX5. **d** Functional mechanism: correlation and enrichment analysis were then used to identify potentially altered pathways that were then confirmed by in vitro and in vivo studies

Identification of causal DNA variants affecting gene expression is complicated as a variety of elements, within the gene and hundreds of kilobases away from the gene, can contribute. Results from the ENCODE (Encyclopedia of DNA elements) and RoadMap Consortia have been particularly useful in this regard for defining enhancer and promoters in a variety of tissues in mice and humans (Box 1, Fig. 3). Once the causal variants or gene have been established, other omics layers can help identify the downstream interactions or pathways. As discussed further below, transcript levels often exhibit poor correlation with protein levels and thus proteomics data are expected to be more proximal to disease mechanisms. Moreover, proteomics techniques such as yeast two-hybrid screens or “pulldown analyses” can be used to identify interacting pathways contributing to disease [27]. For certain disorders, metabolomics can also be used to bridge genotype to phenotype [28].

A good example of a genome first approach is the study by Claussnitzer and colleagues [16] that involved analysis of the FTO locus that harbors the strongest association with obesity (Fig. 3). To identify the cell type in which the causal variant acts, they examined chromatin state maps of the region across 127 cell types that were previously profiled by the Roadmap Epigenomics Project (Box 1). A long enhancer active in mesenchymal adipocyte progenitors was shown to differ in activity between risk and non-risk haplotype. They then surveyed long-range three-dimensional chromatin (Hi-C) interactions involving the enhancer and identified two genes, IRX3 and IRX5, the expression of which correlated with the risk haplotype across 20 risk-allele and 18 non-risk-allele carriers. To identify the affected biologic processes, Claussnitzer and colleagues examined correlations between the expression of IRX3 and IRX5 with other genes in adipose tissue from a cohort of ten individuals. Substantial enrichment for genes involved in mitochondrial functions and lipid metabolism was observed, which suggests possible roles in thermogenesis. Further work using *trans*-eQTL analysis of the FTO locus suggested an effect on genes involved in adipocyte browning. Adipocyte size and mitochondrial DNA content were then studied for 24 risk alleles and 34 non-risk alleles and shown to differ significantly, consistent with an adipocyte-autonomous effect on energy balance. Claussnitzer and colleagues confirmed the roles of IRX2 and IRX5 using experimental manipulation in primary adipocytes and in mice. Finally, the causal variant at the FTO locus was predicted using cross-species conservation and targeted editing with CRISPR-Cas9 identified a single nucleotide variant that disrupts ARID5B repressor binding.

### The phenotype first approach

A different way to utilize omics data to augment our understanding of disease is to simply test for correlations between disease, or factors associated with disease, and omics-based data. Once different entities of omics data are found to correlate with a particular phenotype, they can be fitted into a logical framework that indicates the affected pathways and provide insight into the role of different factors in disease development.

For example, Gjoneska et al. [20] used transcriptomic and epigenomic data to show that genomic and environmental contributions to AD act through different cell types. The authors first identified groups of genes that reflect transient or sustained changes in gene expression and cell populations during AD development. Consistent with the pathophysiology of AD, the transcriptomic data showed a sustained increase in immune-related genes, while synaptic and learning functions showed a sustained decrease. The authors then used chromatin immunoprecipitation and next-generation sequencing (NGS) to profile seven different epigenetic modifications that mark distinct functional chromatin states. They were able to identify thousands of promoters and enhancers that showed significantly different chromatin states in AD versus control. Next, the authors showed that these epigenetic changes correspond to the observed changes in gene expression, and used enrichment analysis to identify five transcription factor motifs enriched in the activated promoters and enhancers and two in the repressed elements. Finally, the authors used available GWAS data to see whether genetic variants associated with AD overlap any of the functional regions they identified. Notably, they found that AD-associated genetic variants are significantly enriched in the immune function-related enhancers but not promoters or neuronal function-related enhancers. This led the authors to suggest that the genetic predisposition to AD acts mostly through dysregulation of immune functions, whereas epigenetic changes in the neuronal cells are mostly environmentally driven.

In another example, Lundby and colleagues [29] used quantitative tissue-specific interaction proteomics, combined with data from GWAS studies, to identify a network of genes involved in cardiac arrhythmias. The authors began by selecting five genes underlying Mendelian forms of long QT syndrome, and immunoprecipitated the corresponding proteins from lysates of mouse hearts. Using mass spectrometry (MS), they then identified 584 proteins that co-precipitated with the five target proteins, reflecting potential protein–protein interactions. Notably, many of these 584 proteins were previously shown to interact with ion channels, further validating the physiological relevance of this experiment. They then compared this list of proteins with the genes located in 35 GWAS loci for common forms of QT-interval variation, and identified 12 genes that overlapped between the two sets. This study provides a mechanistic link between specific genes in some of the GWAS loci to the genotype in question, which suggests a causative link in the locus.

### The environment first approach

In this approach, multi-omics analyses are used to investigate the mechanistic links to disease using an environmental factor such as diet as the variable. To accurately assess environmental or control factors such as the diet in humans is very difficult and so animal models have proven particularly valuable for examining the impact of the environment on disease. Here, we give three examples of multi-omic study designs used to examine the impact of the environment on disease.

One kind of study design is to examine multiple environmental conditions to determine how these perturb physiologic, molecular, and clinical phenotypes. For example, Solon-Biet and colleagues [30] explored the contribution of 25 different diets to the overall health and longevity of over 800 mice. They compared the interaction between the ratio of macronutrients with a myriad of cardiometabolic traits (such as lifespan, serum profiles, hepatic mitochondrial activity, blood pressure, and glucose tolerance) in order to elucidate specific dietary compositions associated with improved health. The ratio of protein to carbohydrate in the diet was shown to have profound effects on health parameters later in life, offering mechanistic insight into how this is achieved.

The second study design seeks to understand the interactions between genetics and the environment. For example, Parks and coworkers [31, 32] recently studied the effects of a high fat, high sucrose diet across about 100 different inbred strains of mice. By examining global gene expression in multiple tissues and metabolites in plasma, they were able to identify pathways and genes contributing to diet-induced obesity and diabetes. In the case of dietary factors, the gut microbiome introduces an additional layer of complexity as it is highly responsive to dietary challenges and also contributes significantly to host physiology and disease. Recent multi-omic studies [31, 33, 34] have revealed an impact of gut microbiota on host responses to dietary challenge and on epigenetic programming.

The third type of study design involves statistical modeling of metabolite fluxes in response to specific substrates. For example, the integration of bibliographic, metabolomic, and genomic data have been used to reconstruct the dynamic range of metabolome flow of organisms, first performed in *Escherichia coli* [35] and since extended to yeast [36, 37] and to individual tissues in mice [38] and humans [39]. Other applications have explored various connections between metabolome models and other layers of information, including the transcriptome [40] and proteome [41–43]. Refinement of these techniques and subsequent application to larger population-wide datasets will likely lead to elucidation of novel key regulatory nodes in metabolite control.

### Integration of data across multi-omics layers

**Fig. 4:**
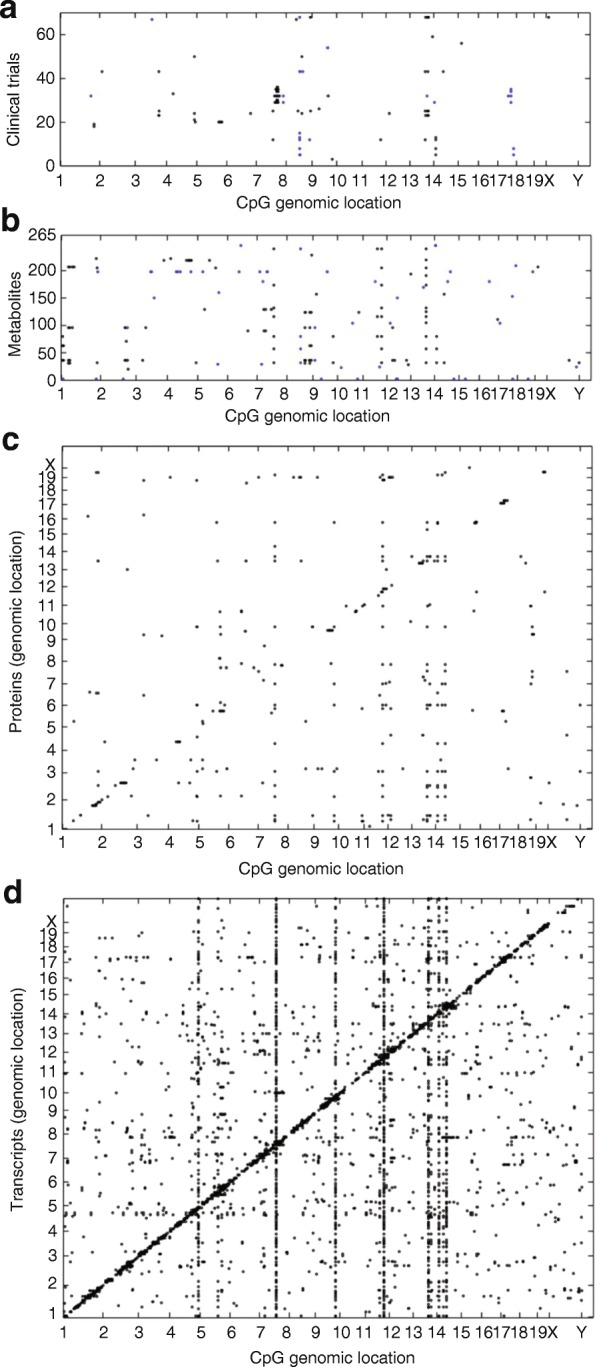
The flow of biologic information from liver DNA methylation to liver transcripts, proteins, metabolites, and clinical traits. A panel of 90 different inbred strains of mice were examined for DNA methylation levels in liver using bisulfite sequencing. CpGs with hypervariable methylation were then tested for association with clinical traits such as **a** obesity and diabetes, **b** liver metabolite levels, **c** liver protein levels, and **d** liver transcript levels. Each *dot* is a significant association at the corresponding Bonferroni thresholds across CpGs with the clinical traits and metabolite, protein, and transcript levels in liver. The genomic positions of hypervariable CpGs are plotted on the *x-axis* and the positions of genes encoding the proteins or transcripts are plotted on the *y-axis*. The positions of clinical traits and metabolites on the *y-axis* are arbitrary. The *diagonal line of dots* observed to be associated with methylation in the protein and transcript data represent local eQTL and pQTL. The *vertical lines* represent “hotspots” where many proteins or transcripts are associated with CpG methylation at a particular locus. Figure taken with permission from [180], Elsevier

A variety of approaches can be used to integrate data across multiple omics layers depending on the study design [44]. Two frequently used approaches involve simple correlation or co-mapping. Thus, if two omics elements share a common driver, or if one perturbs the other, they will exhibit correlation or association (Fig. 4). A number of specialized statistical approaches that often rely on conditioning have been developed. In these approaches a statistical model is used to assess whether each element of the model—for example, a SNP and expression change—contributes to the disease independently versus one being the function of the other. For example, a regression-based method termed “mediation analysis” was developed to integrate SNP and gene expression data, treating the gene expression as the mediator in the causal mechanism from SNPs to disease [45, 46]. Similar approaches have been applied to other omics layers [46, 47]. More broadly, multi-layer omics can be modeled as networks, based on a data-driven approach or with the support of prior knowledge of molecular networks. A practical consideration in multi-omic studies is the correlation of identities of the same objects across omics layers, known as ID conversion. This is performed using pathway databases such as KEGG and cross-reference tables [47]. Ideally, the multi-omics datasets will be collected on the same set of samples, but this is not always possible; GWAS and expression data are frequently collected from different subjects. In such cases, it is possible to infer genetic signatures (eQTL) or phenotypes based on genotypes [48–50].

Investigating the quantitative rules that govern the flow of information from one layer to another is also important when modeling multiple data types. For example, one of the fundamental assumptions behind many of the RNA co-expression networks is that fluctuations in RNA abundance are mirrored by proteins. However, while the tools for effective interrogation of transcriptome are widely available and commonly used, effective interrogation of proteomes at the population level is a relatively new possibility (Box 1). A number of studies have now shown that while levels of many proteins are strongly correlated with their transcript levels, with coincident eQTL and protein QTL (pQTL), the correlations for most protein–transcript pairs are modest [51–58]. The observed discordance of transcript and protein levels is likely to be explained by regulation of translation, post-translation modifications, and protein turnover. Together these studies suggest that RNA may be a good predictor of abundance of only some proteins, identifying groups of genes that confer to this rule and those that do not. In the context of disease oriented research, such studies constitute an important step for creating an analytical framework that will later be applied to interpretation of disease-specific datasets. In addition, especially in context of limited availability of human samples, such studies are useful for choosing among possible experimental approaches.

A key concept of modern biology is that genes and their products participate in complex, interconnected networks, rather than linear pathways [59]. One way to model such networks is as graphs consisting of elements that exhibit specific interactions with other elements [60–64]. Such networks were first constructed based on metabolic pathways, with the metabolites corresponding to the nodes and the enzymatic conversions to the edges [65, 66]. Subsequently, networks were modeled based on co-expression across a series of perturbations with the genes encoding the transcripts corresponding to the nodes and the correlations to the edges [67–69]. In the case of proteins, edges can be based on physical interactions, such as those identified from global yeast two-hybrid analyses or a series of “pulldowns” [27]. Networks can also be formed based on genomic interactions captured by HiC data [70, 71], and physical interactions can also be measured across different layers, such as in ChIP-Seq, which quantifies DNA binding by specific proteins.

For studies of disease, co-expression networks can be constructed based on variations in gene expression that occur among control and affected individuals separately [72–74]. Comparison of network architecture between control and disease groups allows the identification of closely connected nodes (“modules”) most correlated with disease status. In general, co-expression or interaction networks are “undirected” in the sense that the causal nature of the interactions is unknown. Interaction networks can be experimentally tested, although the high number of suggestive interactions identified in each study makes indiscriminate testing prohibitive. If genetic data, such as GWAS loci for disease or eQTLs for genes, are available it may be possible to infer causality using DNA as an anchor [75–77]. Such integration of genetic information with network modeling has been used to highlight pathways that contribute to disease and to identify “key drivers” in biologic processes [72–74, 78]. For example, Marbach and colleagues [79] combined genomics, epigenomics, and transcriptomics to elucidate tissue-specific regulatory circuits in 394 human cell types. They then overlaid the GWAS results of diseases onto tissue-specific regulatory networks in the disease-relevant tissues and identified modules particularly enriched for genetic variants in each disease. In another example, Zhang and coworkers [64] examined transcript levels from brains of individuals with late onset AD and analyzed co-expression and Bayesian causal modeling to identify modules associated with disease and key driver genes important in disease regulatory pathways. Together, these studies illustrate how network analysis can be used to narrow down the focus of disease research into specific functional aspects of particular cell types or tissues, considerably facilitating downstream mechanistic efforts and hypothesis generation.

## Current challenges and future directions

### Reference populations and phenotyping

Insights gained from omics approaches to disease are mostly comparative. We compare omics data from healthy and diseased individuals and assume that this difference is directly related to disease. However, in complex phenotypes both “healthy” and “disease” groups are heterogeneous with respect to many confounding factors such as population structure, cell type composition bias in sample ascertainment, batch effects, and other unknown factors.

One strategy to overcome the heterogeneity associated with any human population is the “reductionist approach”, which aims to match as closely as possible groups of patients and controls to eliminate many of the environmental factors from this comparison. The problem with this approach is two-fold. First, we do not know about every possible confounding factor, and thus we can only account for known sources of variation (for example, sex, BMI, age, and diet in metabolic disease). And second, insight is limited to the variable factors included in the study, which might not apply when considering the whole spectrum of disease population or might be entirely secondary to a factor that was excluded. In contrast, an integrative omics approach often relies on a “holistic” view, which attempts to interrogate a sufficiently large number of individuals and incorporate the many sources of variability into statistical models. The differences observed between disease and healthy state are then compared to identify factors that have a larger contribution to the disease. Thus, a crucial aspect for success of omics studies is the collection of large datasets that accurately capture sources of variance in the background population (or “healthy” individuals). Collection of such data is becoming feasible. The increasing popularity of lifestyle tracking devices and social media has created an unprecedented opportunity for studying environmental factors that contribute to disease development and progression on a large scale, and further integration with omics data may provide additional guidance for personalization of treatment. A recent study used an integrative omics approach in personalized nutrition. Zeevi et al. [80] used combinatorial analysis of questionnaire data, microbiome data, plasma parameters, and a meal diary among 800 individuals to predict postprandial glycemic index, which was used to provide accurate information on dietary regimens to improve metabolic homeostasis.

The power of omics approaches, and their greatest challenge, will be the ability to integrate multiple axes of variance into background models, rather than researching age, sex, time, and population specific instances. Thus, we expect future application of omics technologies to focus on understudied groups, particularly in the sex specificity context, to fill substantial gaps in our knowledge and lead to the development of more informative models of biological context of disease. Sex is one of the major determinants of biological function, and most diseases show some extent of sex dimorphism [81]. Thus, any personalized treatment approaches will have to take sex into account. Indeed, the National Institutes of Health has recognized that need recently and explicitly drives biomedical research towards sex-balanced studies (http://grants.nih.gov/grants/guide/notice-files/NOT-OD-15-102.html).

Human populations that can be interrogated at multiple omics levels or examined under a variety of environmental conditions are proving particularly powerful. For example, the MuTher study [82], consisting of several hundred female twins from the UK, has been evaluated globally at the genome, transcriptome, metabolome, and microbiome levels. Data from this study have yielded a variety of important conclusions, including insights into the genetic control of molecular traits, novel pathways involved in metabolic syndrome, and the heritability of gut microbiota [78]. Twin studies are particularly powerful in their ability to accurately estimate heritability of traits. Another human reference population is the Metabolic Syndrome In Man (METSIM) cohort of about 10,000 Finnish men aged 45–65 years from the Kuopio region in Finland. As with the MuTher population, METSIM individuals have been characterized clinically for a variety of metabolic and cardiovascular traits at the genomic, transcriptomic, and metabolomics levels [83–85]. The METSIM population is especially appealing given the broad spectrum of metabolic measurements and subsequent follow-ups.

### Technological advances and resolution

While great technological progress has been made, we believe routine implementation of omics data on a population scale will likely require further improvements in data acquisition, analysis, and cost-effectiveness. One area in particular which has gained substantial attention recently is the role of the gut and other microbes in the maintenance of homeostasis. The microbiome has been shown to alter many aspects of host physiology, from obesity [86, 87] to cognition [88]. Improvements in MS acquisition and analysis platforms for bacterial-derived compounds will draw many additional links between microorganism composition/activity and overall health status and provide more and more accurate proteomics and protein modification data. Instrumentation for global acquisition of proteomics data, comparable to the resolution scale of RNA-Seq, will likely allow for defined pathway interrogation and set the stage for comprehensive examination of vital cellular functions, such as signaling pathways. Phosphoproteomics, in particular, has been utilized to elucidate novel signaling mechanisms [66]. Beyond the phosphoproteome, omics analyses have drawn notable links between human disease and the genetic control of global glycosylation [68], ubiquitination [67, 69], and many other protein modifications. Continued improvements in these approaches will further our understanding of protein functions.

Recent technological advances have also allowed for NGS to be performed on single cells [89], an area which has received considerable attention [90]. RNA-Seq using a single-cell approach has shown substantial heterogeneity of cell types in various tissues and elucidated novel cell populations [91, 92]. Beyond sequencing the transcriptome of single cells, this technology has been extended to the genome [93] and DNA methylome [94–96]. Bisulfite sequencing of single cells has shown substantial variations in the pattern of DNA methylation across cells residing in the same tissues, presenting a unique opportunity to explore combinatorial roles for differing cell types presented with a similar “environmental exposure”. Single cell analysis also allows quantification and identification of the omics changes that are observed at the tissue level that are attributable to changes in cell type composition, rather than changes in the respective omics profile of specific cell types—an important aspect of disease physiology.

### Analytical challenges

One obvious advantage of large omics datasets is their enduring availability—once the data are collected, they can be reanalyzed with multiple approaches over and over again. Thus, development of statistical methods to extract more information from existent data types is an important part of the omics field. While every omics field presents specific challenges in terms of technical artifacts, a common analytical challenge to all omics fields is distinguishing causal changes from reactive ones in the context of disease. This is particularly difficult because of the correlative nature of each dataset, and potentially impossible if relying on one omics data type collected at one time point, such as the expression in tissues in healthy and diseased individuals postmortem. Development of approaches to differentiate causal changes versus correlative changes should address two questions: first, identifying the variation that causes or drives the association with phenotype; and second, elucidating whether that variation precedes the trait or occurs as a result of it. Notably, genomic changes associated with disease are presumed to precede it, and therefore the question of causality in GWAS loci comes down to identifying the precise variant driving the correlation. Several approaches have been developed to identify drivers of the correlation signals in genomic or transcriptomic data [11, 97, 98]. However, when the drivers of correlation are identified, with the exception of genomics, differentiating causality from correlation based on omics analysis remains an open question. We envision that development of better statistical methods, overlaying of multiple coordinated data types, prospective studies in humans, and time-course studies in animal models will help narrow the candidates to sufficiently small numbers that can efficiently be tested in cellular and animal models. Yet, the final proof of causation that relates a particular change to a particular phenotype is likely, for the foreseeable future, to rely on molecular studies in vivo and in vitro.

### Conceptual shift

The future of medical research envisions personalized treatments, prospective tracking of individual health indicators, and a focus on preventive measures that integrate into our way of life. A proof of concept study [99] shows that prospective tracking of health with multiple omics approaches could highlight indicators of disease prior to the development of disease, and that beneficial changes in lifestyle might help to prevent it. Furthermore, applications of omics technologies within a clinical setting can be used in personalized medicine, guided by genome sequence. A poster-child example of such has been implemented through the Vanderbilt PREDICT project [100], whereby genotyping information is gathered and referenced to patient data throughout the treatment process to identify individual variants that affect clinical outcomes.

As the cost of omics analyses continues to decrease, more types of high throughput data can guide individualized treatment regimens and be integrated into the clinic. However, such undertaking also poses significant challenges. The ever-growing amount and sophistication of our knowledge, combined with the sheer quantity of data and technical expertise required for comprehensive collection and analysis of multi-omics data, are far from trivial. No one research group on their own can handle multi-scale omics data generation, development of analytical methodology, adaptation of those methods to specific disease, and functional follow-up, let alone repeating this process for multiple diseases and integrating between them. To be efficient and translatable in the clinic, such undertakings necessitate coordinated efforts of many groups, each providing its own expertise or resource, as reflected by the formation of large consortia. Some consortia efforts (e.g., ENCODE) focus on investigating a series of omic data on coordinated sets of samples, providing invaluable insight into the basic biological properties reflected by these data, and development of rigorous analytical frameworks that can be then applied or adapted to other datasets. Other consortia may focus on tissue specificity [101], particular disease, or resource development.

Effective and sensible use of publicly available data requires a standard, easily communicable terminology and methodology in all aspects of data collections and analysis—sometimes even at the expense of precision or optimization. Common usage of omics technologies necessitates standardization to allow sufficient integration across studies, an area which becomes increasingly difficult with greater variability and complexity of measurement. For example, RNA-Seq expression studies are only comparable if the same genome version, transcript annotation, and quantification tools are used for all datasets, while new versions of these are published on a regular basis. For this reason, consortia provide both a large body of data but also detailed analysis pipelines that can be replicated for other datasets with minimal effort. Standardization becomes particularly challenging when measuring various phenotypes and relating from one study to another. Suggestions have been made to apply standardization across measured phenomes. For example, various high-throughput biologic assays have been developed to screen mutagenized mice [102–104] or zebrafish [105]. Such assays can be thought of as “subphenotypes” of disease, likely to be much less genetically complex (and, therefore, easier to dissect) than the disease itself. Additional efforts have been made to apply a “phenomics” approach to understand human disease [106]. We believe that further improvement in streamlining the analysis of specific data types, and development of a gold standard for analysis flow, will facilitate new discoveries and shorten the time taken from the generation of data to publication and translation to clinics. Notably, this facet of omics research is particularly vulnerable not only to technical problems (e.g., use of different protocols and analysis pipelines, changes in data ID numbers, lack of standard nomenclature, etc.), but also to social behavior that drives cutting edge research. A glaring example of this psychological gap was recently demonstrated by the “data parasites” editorial in a prominent medical journal [107], and the prompt stormy reaction in scientific and social outlets that followed [108–110]. This incident highlights that successful application of the omics approach does not depend solely on technical factors but requires a serious conceptual shift in research paradigm for many researchers and clinicians, and potentially in our approach to medical and scientific training and performance evaluation.

## Abbreviations

AD: Alzheimer’s disease
eQTL: Expression quantitative trait loci
GWAS: Genome-wide association studies
MS: Mass spectrometry
NGS: Next-generation sequencing
pQTL: Protein quantitative trait loci
SNP: Single-nucleotide polymorphism
